# Metabolomic Approaches to Explore Chemical Diversity of Human Breast-Milk, Formula Milk and Bovine Milk

**DOI:** 10.3390/ijms17122128

**Published:** 2016-12-17

**Authors:** Linxi Qian, Aihua Zhao, Yinan Zhang, Tianlu Chen, Steven H. Zeisel, Wei Jia, Wei Cai

**Affiliations:** 1 Shanghai Institute for Pediatric Research, Xinhua Hospital, School of Medicine, Shanghai Jiao Tong University, 1665 Kongjiang Road, Shanghai 200092, China; microtuble@sina.com; 2 Shanghai Key Laboratory of Pediatric Gastroenterology and Nutrition, Xinhua Hospital, School of Medicine, Shanghai Jiao Tong University, 1665 Kongjiang Road, Shanghai 200092, China; 3 Center for Translational Medicine, Six People’s Hospital, Shanghai Jiao Tong University, 600 Yishan Road, Shanghai 200233, China; zhah@sjtu.edu.cn (A.Z.); zhyn@sjtu.edu.cn (Y.Z.); chentianlu@sjtu.edu.cn (T.C.); 4 Nutrition Research Institute, University of North Carolina, Chapel Hill, Kannapolis, NC 28081, USA; steven_zeisel@unc.edu

**Keywords:** metabolomics, GC-TOFMS, UPLC-QTOFMS, fatty acids, tricarboxylic acid intermediates, amino acids, carbohydrate

## Abstract

Although many studies have been conducted on the components present in human breast milk (HM), research on the differences of chemical metabolites between HM, bovine milk (BM) and formula milk (FM) is limited. This study was to explore the chemical diversity of HM, BM and FM by metabolomic approaches. GC-TOFMS and UPLC-QTOFMS were applied to investigate the metabolic compositions in 30 HM samples, 20 FM samples and 20 BM samples. Metabolite profiling identified that most of the non-esterified fatty acids, which reflected the hydrolysis of triglycerides, were much more abundant in HM than those in FM and BM, except for palmitic acid and stearic acid. The levels of tricarboxylic acid (TCA) intermediates were much higher in FM and BM than those in HM. Each type of milk also showed its unique composition of free amino acids and free carbohydrates. In conclusion, higher levels of non-esterified saturated fatty acids with aliphatic tails <16 carbons, monounsaturated fatty acids and polyunsaturated fatty acids and lower levels of TCA intermediates are characteristic of HM, as compared with FM and BM. The content of non-esterified fatty acids may reflect the hydrolysis of triglycerides in different milk types.

## 1. Introduction

Human breast milk (HM) is the primary source of nutrition for healthy infant growth. It not only contains normal nutrients, such as carbohydrates, fats, proteins, minerals and vitamins, but also various biologically-active constituents, including antimicrobial substances, growth factors, cytokines, immunoglobulins and specific immune cells [1]. The benefits of breastfeeding are widely accepted [2,3,4,5,6,7,8,9], and HM is considered to be the gold standard for formula milk (FM). However, exclusive breastfeeding is not always possible, and breastfeeding is contraindicated for certain medical conditions [10]. Consequently, there have been many attempts to produce FM that resembles HM as closely as possible. Thus, the characterization of HM composition, in relation to infant nutrition, is important for the improvement of FM. However, the composition of HM is quite variable and is influenced by numerous maternal, environmental and genetic factors [11,12,13]. Attempts to more accurately mimic HM depend on the understanding of the complexity of the nutritive and non-nutritive components in HM.

Whereas much research has been performed on the highly abundant components of HM, a complete understanding of all of its components is limited, particularly of the metabolites present in HM. Only a few preliminary metabolomic studies using nuclear magnetic resonance (NMR) or gas chromatography-mass spectroscopy (GC-MS) have been previously conducted on HM, and the number of metabolites detected by these studies is modest due to their relatively low sensitivities [14,15,16,17,18]. Several other studies have been conducted targeting specific chemical classes, such as oligosaccharides and sphingomyelins [19,20]. In recent years, the development of metabolomic technological tools has been improved rapidly and allowed us to cover the global metabolic profiles of biological samples. Mass spectrometry (MS)-based metabolomics offer highly selective and sensitive analyses with the potential to identify a high abundance of metabolites, especially in combination with GC-MS and liquid chromatography-mass spectrometry (LC-MS) [21,22]. Moreover, sample pre-treatment protocols have been optimized to capture as many metabolites as possible using a single extraction phase [23].

In this study, we tested the potential of the MS-based methodology to investigate the metabolic composition of HM using a single-phase approach. For the sake of comparison, some commercially-available FM and bovine milk (BM) were also analyzed. We applied a comprehensive metabolite profiling approach using gas chromatography-time-of-flight mass spectrometry (GC-TOFMS) and ultra-performance liquid chromatography-quadrupole-time-of-flight mass spectrometry (UPLC-QTOFMS) to systematically compare a large variety of substances, such as carbohydrates, amino acids, organic acids, amines, fatty acids and bile acids, in milk types and to interpret the differences between them. The bioresources in the NIST library 2005 and Human Metabolome Database (HMDB) were used in this study [24,25]. To the best of our knowledge, no global MS-based assays have been previously used to compare the metabolic profiles of HM, FM and BM.

## 2. Results

Self-reported participant perinatal history data, including age, multiparity, prepregnancy body mass index, gestational weeks, C-section history, baby gender and birth weight, are shown in Table 1; 46.7% women delivered their infants through C-section. Approximately equal numbers of male and female infants were born. Throughout the breastfeeding period, all infants were healthy and did not experience any nutrition-related illnesses.

**Figure 1:**
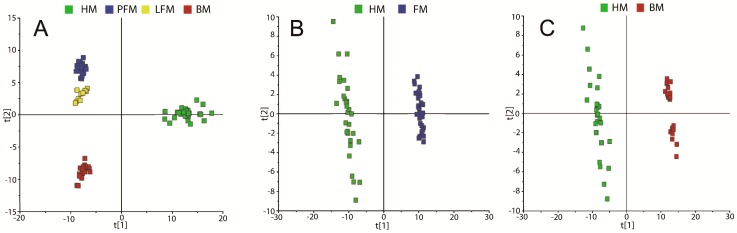
The scores plots of PLS-DA model constructed with annotated metabolites. (**A**) The scores plot of PLS-DA model discriminating HM, LFM, PFM and BM; (**B**) the scores plot of PLS-DA model discriminating HM and FM; (**C**) the scores plot of PLS-DA model discriminating HM and BM. Each component axis is denoted as t[x] on the score plots where x is the component number. HM, human breast milk; LFM, liquid formula milk; PFM, powdered formula milk; FM, formula milk; BM, bovine milk.

Two hundred and eighty metabolites were annotated from the detected spectral features from GC-TOFMS and UPLC-QTOFMS, using reference standards, as well as the available database (NIST library 2005 and HMDB). To distinguish HM from BM, powered formula milk (PFM) and liquid formula milk (LFM), PLS-DA was performed with the annotated metabolites (Figure 1A). There was a clear separation between HM and the other milk types, reflecting the unique metabolite profile of HM. As expected, the metabolic patterns of PFM and LFM were very similar, and they were distinct from that of HM and BM. Thus, PFM and LFM were merged as one group, referred to as FM, for later analysis. Furthermore, the PLS-DA model of 280 metabolites demonstrated distinctly different metabolite profiles between HM and FM (*R*^2^(X) = 0.379, *R*^2^(Y) = 0.998, *Q*^2^ = 0.985) using one predictive component and two orthogonal components (Figure 1B). Analogously, a distinct separation was observed between the metabolite profiles of HM and BM (*R*^2^(X) = 0.44, *R*^2^(Y) = 0.998, *Q*^2^ = 0.992), which is indicative of the distinct metabolic profile of HM (Figure 1C). The significant permutation test (*p* < 0.01) confirmed the results of PLS-DA analysis.

**Figure 2:**
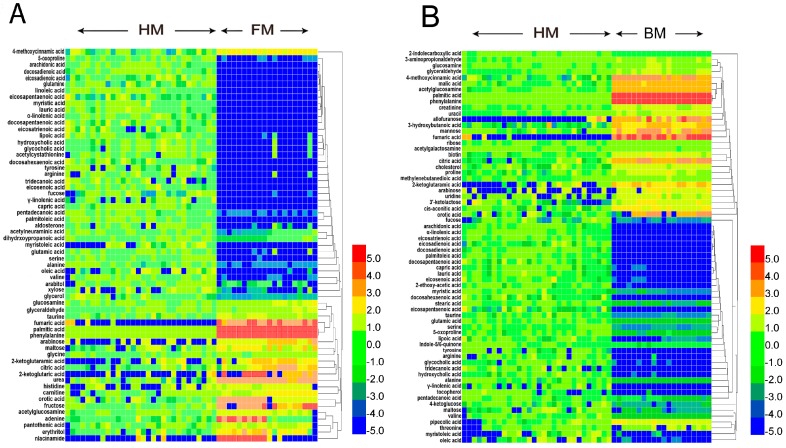
Heatmaps of hierarchical cluster analysis comparing the metabolite changes among HM, FM and BM. The heatmaps were generated using the differential metabolites (variable importance in the projection (VIP) >1, *p* < 0.05) for the comparison of FM vs. HM (**A**); and BM vs. HM (**B**). The heatmap graphically represents individual changes of ion intensity. The color red represents the relatively higher abundance, and the color blue represents the relatively lower abundance of each metabolite. The fold of change was normalized by log2 transformation. Hierarchical clustering agglomeration was performed on the identified metabolites using the Pearson correlation as the distance metric. HM, human breast milk; FM, formula milk; BM, bovine milk.

Based on the variable importance in the projection (VIP) value (>1) and the Mann–Whitney U test *p*-value (<0.05), 61 and 66 significantly differential metabolites were identified in FM and BM, respectively, compared to HM (Tables S1 and S2). These metabolites include a large variety of substances, such as carbohydrates, amino acids, organic acids, amines, fatty acids and bile acids. Heatmap plots were generated for the comparison of FM and BM vs. HM, according to the ion intensities of the metabolites (Figure 2). The differential metabolites were then clustered according to their Pearson correlation coefficients. It was found that the characteristic metabolites in HM segregated from FM and BM were mainly clustered into four groups: (1) non-esterified fatty acids (NEFAs); (2) free amino acids (FAAs); (3) tricarboxylic acid (TCA) intermediates; (4) free carbohydrates.

**Figure 3:**
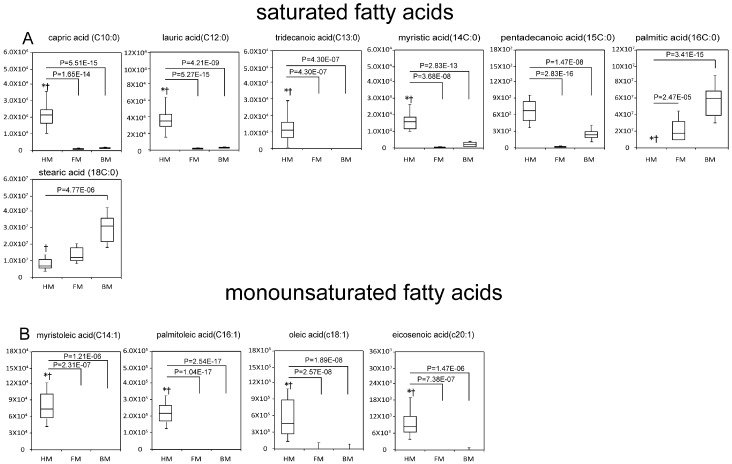

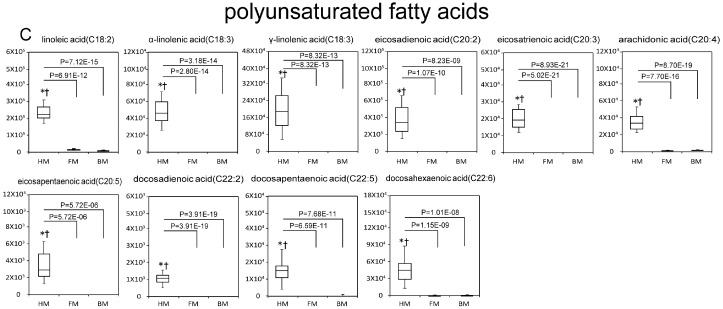
Comparison of the relative abundances of saturated fatty acids (**A**), monounsaturated fatty acids (**B**) and polyunsaturated fatty acids (**C**) among HM, FM and BM. The Y-axis represents the normalized peak area. Boxes contain the 25%–75% measurements for each group, and whiskers cover the 5%–95% measurements. The horizontal lines within the box represent the median values. * *p* < 0.05 vs. FM; † *p* < 0.05 vs. BM. Kruskal–Wallis H test followed by Bonferroni-corrected post-hoc test. HM, human breast milk; FM, formula milk; BM, bovine milk.

The complexity of NEFAs, which reflects the hydrolysis of triglycerides, was found to be indicative of HM. These fatty acids include saturated fatty acids (SFAs), monounsaturated fatty acids (MUFAs) and polyunsaturated fatty acids (PUFAs). As revealed by our study, the non-esterified SFAs were much less abundant in FM and BM than those in HM, except for palmitic acid (16C:0) and stearic acid (18C:0) (Figure 3A). These two SFAs contain longer aliphatic tails and were observed to be more abundant in BM than those in HM. The non-esterified myristoleic acid (14C:1), palmitoleic acid (16C:1), oleic acid (C18:1) and eicosenoic acid (C20:1), which belong to MUFAs, were also much less abundant in FM and BM than those in HM (Figure 3B). Two essential PUFAs include linoleic acid (C18:2) and alpha-linolenic acid (C18:3). Other major PUFAs, which can be converted from essential fatty acids, include eicosapentaenoic acid (C20:5), docosapentaenoic acid (C22:5) and docosahexaenoic acid (C22:6) of the omega-3 series; and gamma-linolenic acid (C18:3), eicosadienoic acid (C20:2), eicosatrienoic acid (C20:3), arachidonic acid (C20:4) and docosadienoic acid (C22:2) of the omega-6 series. The non-esterified forms of all these PUFAs were scarce in FM and BM (Figure 3C).

**Figure 4:**
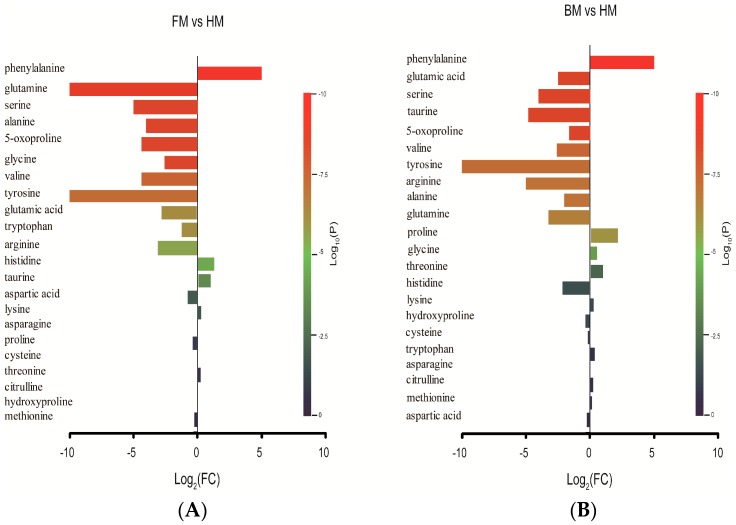
Bar plot of the amino acid changes for the comparison of FM vs. HM (**A**); and BM vs. HM (**B**). A fold change (FC) value for each amino acid (X) was calculated as follows: X_fold change_ = (X_level in FM or BM_/X_level in HM_). Each bar representing an FC value was colored to indicate its corresponding *p*-value and thereby to specify the statistical significance of the difference (see the color scale). The FC value was normalized by log2 transformation, and the *p*-value was normalized by log10 transformation. HM, human breast milk; FM, formula milk; BM, bovine milk.

Another prominent cluster separating HM from FM and BM was FAAs. Among the identified FAAs, the levels of phenylalanine, histidine and taurine, which are essential amino acids for infant growth, were significantly higher in FM than those in HM. The levels of 5-oxoproline, alanine, arginine, glutamic acid, glutamine, glycine, serine, tyrosine and valine were less abundant in FM than those in HM (Figure 4A). In BM, the levels of phenylalanine and proline were observed to be higher than those in HM. 5-oxoproline, alanine, arginine, glutamic acid, glutamine, serine, taurine, tyrosine and valine were less abundant in BM than those in HM (Figure 4B). In comparison, the top significant FAA, which was elevated in both FM (*p* = 1.96 × 10^−11^) and BM (*p* = 1.82 × 10^−11^) was phenylalanine. Taurine and histidine, the other two elevated FAAs in FM, were found to be decreased or non-differential in BM relative to HM. Among the decreased FAAs, the most significant one was glutamine in FM (*p* = 8.24 × 10^−10^) and glutamic acid in BM (*p* = 2.83 × 10^−9^). Most of the FAAs decreased in FM were also lower in BM.

**Figure 5:**
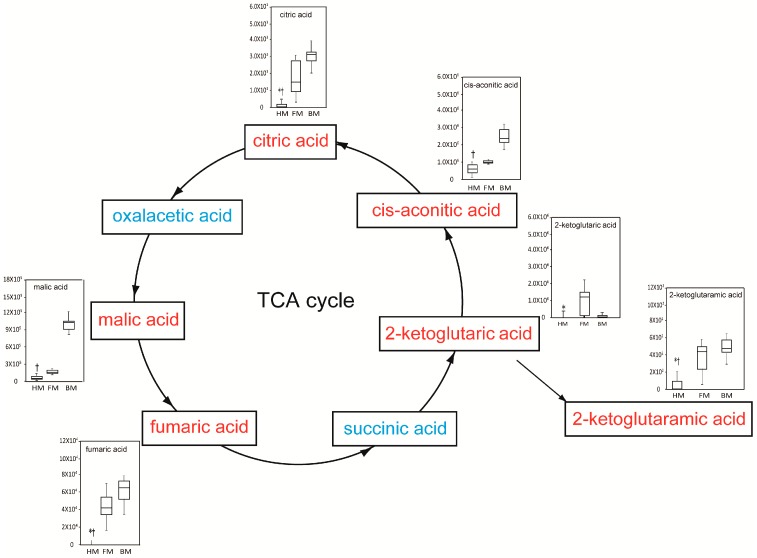
Bar plot of tricarboxylic acid (TCA) intermediates that significantly differed among HM, FM and BM. Blue labeling represents no significant difference among milk types. Red labeling represents a significant difference among milk types. The *Y*-axis represents the normalized peak area. Boxes contain the 25%–75% measurements for each group, and whiskers cover the 5%–95% measurements. The horizontal lines within the box represent the median values. * *p* < 0.05 vs. FM; † *p* < 0.05 vs. BM. Kruskal–Wallis H test followed by Bonferroni-corrected post-hoc test. HM, human breast milk; FM, formula milk; BM, bovine milk.

The levels of TCA intermediates, including 2-ketoglutaric acid, 2-ketoglutaramic acid, citric acid and fumaric acid, were 3.23–27.54-fold higher in FM than those in HM. 2-ketoglutaramic acid, cis-aconitic acid, malic acid, citric acid and fumaric acid were 3.08–33.53-fold higher in BM than those in HM. Oxaloacetic acid and succinic acid showed no significant difference among HM, FM and BM (Figure 5).

**Figure 6:**
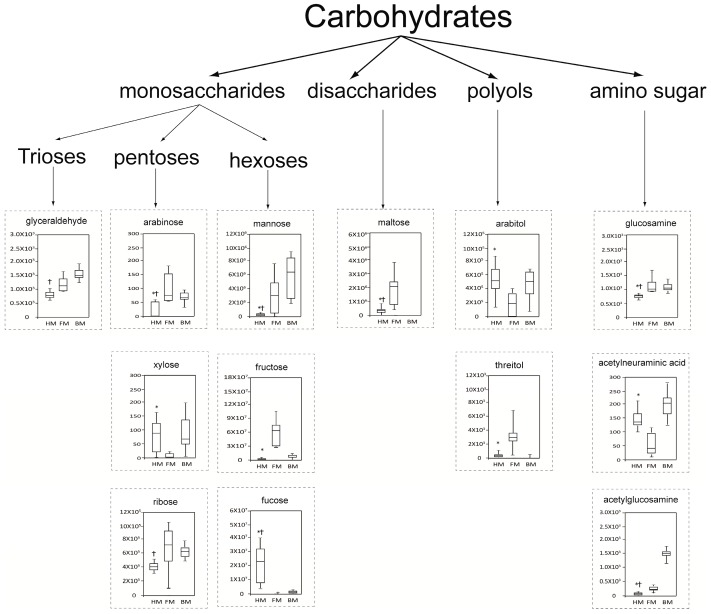
Bar plot of carbohydrates that significantly differed among HM, FM and BM. The Y-axis represents the normalized peak area. Boxes contain the 25%–75% measurements for each group, and whiskers cover the 5%–95% measurements. The horizontal lines within the box represent the median values. * *p* < 0.05 vs. FM; † *p* < 0.05 vs. BM. Kruskal–Wallis H test followed by Bonferroni-corrected post-hoc test. HM, human breast milk; FM, formula milk; BM, bovine milk.

The difference of carbohydrate profiles among HM, FM and BM is complex (Figure 6). Each milk type showed a unique profile of monosaccharides, disaccharides, polyols and amino sugars. The abundance of arabinose, mannose, glucosamine and acetylglucosamine was lowest in HM as compared to FM and BM; whereas the abundance of fucose was highest in HM as compared to FM and BM. Fructose, maltose and threitol showed highest abundance in FM as compared to HM and BM. Lactose, which was the main energy supplier in milk, did not differ among HM, FM and BM. Fructose is the sweetest monosaccharide (1.8-fold relative to sucrose) among the detected sugars in three milk types; its abundance was 29.63-fold higher in FM than that in HM.

Several vitamins exhibited considerably higher abundance in FM as compared to HM. The level of niacinamide, the amide of niacin (vitamin B3), was 31.38-fold higher in FM than that in HM; however, its level was comparable between HM and BM. The level of adenine (vitamin B4) was 20.15-fold higher in FM than that in HM; however, its level was also comparable between HM and BM. The level of tocopherol (vitamin E) in FM was not significantly different from that in HM, but its level in BM was only 1% of the level in HM (Tables S1 and S2). From the comparison, we obtained the clue that these three vitamins are fortified in our FM samples.

## 3. Discussion

In the current study, we comprehensively determined the metabolite content of HM, FM and BM by using both GC-TOFMS and UPLC-QTOFMS. Two hundred and eighty metabolites were annotated in the collected milk samples. The number of identified metabolites in the present study was much more than that reported by two previous studies, which was 43 metabolites [17] and 36 metabolites [18], respectively. Through multivariate and univariate statistical analyses, a unique metabolic profile was observed for HM. We identified metabolites whose abundances were distinct between HM and FM (or BM) and thus could represent the metabolites that might have specific physiological roles for the developing neonates.

Lipids are important nutrients present in HM, as they are major energy providers for growing infants and supply approximately 45%–55% of total energy [26]. They also provide lipid-soluble vitamins and essential PUFAs. High abundances of PUFAs are present in the retina and brain of infants, and their abundances are proposed to increase steadily during the first year of life [27]. Moreover, a number of studies demonstrated that the supply of appropriate PUFAs in early life is associated with better visual and cognitive development [28,29]. Due to these benefits, several health authorities have defined recommendations and guidelines for regulating the appropriate supplementation of PUFAs in infant foods [30]. As we know, most fatty acids in the milk normally exist as esters, and the PUFAs supplements in FM are provided in the form of triglycerides [31]. The content of NEFAs represents a small fraction (≤1%) of total fatty acids in HM, and the percentage of NEFAs in the milk relies on the degree of hydrolysis [32]. However, the NEFAs play specific roles in the absorption of fatty acids in HM. The unsaturated fatty acids (UFAs) and medium-chain SFAs are readily absorbed as the non-esterified form, while long-chain SFAs, such as palmitic acid and stearic acid, are readily absorbed as 2-monoglycerides in humans [33]. The bile salt-dependent lipolytic activity in HM has the potency to release UFAs and medium-chain SFAs from triglyceride at the sn-1 and sn-3 positions to enhance their absorption rate [34]. However, the long-chain SFAs tend to remain esterified to glycerol at the sn-2 position in HM [33]. The present study showed that HM contains higher amounts of non-esterified MUFAs and PUFAs than FM and BM. The non-esterified SFAs with aliphatic tails <16 carbons were more abundant in HM than those in FM and BM. Palmitic acid and stearic acid with aliphatic tails ≥16 carbons were less abundant in HM than those in FM and BM. All of the characteristics of NEFAs distribution in HM contribute to the better absorption of these nutrients by the infant gut. In addition to their unique nutrition roles, NEFAs of HM also have anti-parasite and anti-viral properties. The fatty acids, released during hydrolysis of milk triglycerides, are responsible for the killing of *G. lamblia* by HM [35]. Increasing concentrations of non-esterified PUFAs in HM might reduce the risk of mother-to-child HIV transmission [36]. More research is needed to further explore the physiological role of NEFAs in HM and their nutritional indication in FM.

Amino acids represented another metabolite cluster, which showed significant differences among the distinct types of milk. The content of FAAs represents a small fraction (3%–5%) of total amino acids in HM, and the percentage of FAAs in FM relies on the degree of hydrolysis [37]. WHO standards do not specify the content of FAAs in FM [38], and the concentrations of FAAs present in FM are not typically provided in the manufacturers’ specification. However, they are considered an important nitrogen source for growing infants and are more readily absorbed than protein-derived amino acids [39]. Some FAAs, such as glutamic acid and glutamine, are important for the differentiation and proliferation of intestinal epithelial cells [40]. Free glutamic acid in FM was reported to confer satiety to the infants and control the milk intake [41]. In our study, multiple FAAs were less abundant in both BM and FM as compared to HM; however, some of the essential ones, such as histidine and taurine, were obviously balanced in FM samples. Phenylalanine was the only essential amino acid that was elevated in both FM and BM. The difference of FAA profiles between HM and FM revealed by the present study largely agreed with that shown by Scano’s study [17], except for tyrosine, which showed opposite results. Due to the unbounded nature, FAAs can be sensed by receptors in the taste-goblet and impact the flavor of the milk [42]. Each free amino acid has its unique flavor [43]. For example, glutamic acid may be “meaty, salty, bitter and complex”, and valine may be “flat to sweet; sometimes sulfurous, meaty or sweet”. The combination of FAAs contributes to the taste profile of different milk types. Thus far, the research on the FAAs in FM and HM was limited, and the physiological role of FAAs and their impacts on the flavor of different milk types need to be further studied.

The cluster of TCA intermediates also showed a significant difference among FM, HM and BM. TCA intermediates are traditionally considered providers of carbon skeletons for anabolism and reducing power sinks for the respiratory chain [44]. More recently, they have gained recognition as important signaling molecules for the regulation of gene expression and cell differentiation [45]. The cellular TCA intermediates are presumably of importance in the process of post-translational modification and are implicated in metabolic remodeling through the control of DNA demethylation and protein degradation [46,47]. Moreover, TCA intermediates might also play important roles in promoting and regulating cell differentiation. For example, cellular citric acid has been shown to alter glycolytic and oxidative metabolism in mesenchymal cells, which underlies the differentiation of mesenchymal cells into functional osteoblasts [48,49]. Likewise, TCA intermediates have been implicated in the alteration of gene expression during the differentiation of mouse 3T3L1 cells to adipocytes [50,51]. Compared to HM, the abundance of TCA intermediates was much higher in FM and BM. This difference was also revealed by Scano’s study, with malic acid as an index [17]. Based on the literature, the effect of dietary intake of TCA intermediates was only studied in adults, and the results showed that the intake has no effect with regard to improving the ability of aerobic exercise [52]. The lack of evidence restrained us from understanding the role of the high abundance of TCA intermediates in BM and the impact of drinking TCA intermediate-rich FM on infant growth. Nevertheless, it is still plausible to speculate that the high concentration of TCA intermediates in BM may be an adaptation for the ruminant growth via metabolic remodeling. Whether the overconsumption of TCA intermediates is safe for the growth of human infants needs further investigation in the future.

Oligosaccharides are the third most abundant solid component in HM and BM [53]. Oligosaccharides are typically cleaved into smaller monosaccharides and amino sugars by glycoside hydrolases. However, some oligosaccharides are generally not digestible by the infant and remain largely intact until they reach the colon [54]. The free sugars and amino sugars in milk could partially reflect their proportions in glycan and the susceptibility of their glycosidic bonds to glycoside hydrolases [55]. Previous studies identified remarkable differences in the structures and relative abundances of the different oligosaccharide types between HM and BM. For example, high fucosylation was observed as general feature of human milk oligosaccharides (HMOs), whereas bovine milk oligosaccharides (BMOs) were reported almost lacking in fucosylation [56]. Another feature of HMOs is the lower composition of mannose as compared to BMOs, and most of the mannose residues reside in the core of glycan and are presumably resistant to glycoside hydrolases [56]. It was found HM contained higher free fucose and lower free mannose relative to FM and BM in the present study, and this trait could be explained by the abundance and structural difference of oligosaccharides between HM and BM. The cleaved fucose serves as prebiotics to stimulate the growth of beneficial intestinal bacteria. For example, fucose could bolster the beneficial species of the gut microbiota, like the *Ruminococcaceae* and *Bacteroides* species, thus maintaining their beneficial functions, such as colonization resistance to opportunistic pathogen [57].

Intriguingly, the present study showed that FM contained fructose 30.63-times as much as that in HM. The overconsumption of fructose is associated with adverse metabolic changes that are harmful to infants [58,59,60]. Fructose is even prohibited to be used instead of lactose as a carbon supplier in FM [61]. The limitation of our study was that the results lacked absolute quantitation, and only the relative quantity of each metabolite was compared between milk samples. Further study should be conducted to determine the absolute quantity of fructose in FM and to investigate their sources in FM. We postulated that the fructose in our FM samples may be derived from fructo-oligosaccharides, which are routinely supplemented as prebiotics [17,62]. Actually, some polyols can also be introduced into FM by prebiotics supplementation. Our study found that FM contained higher threitol relative to HM. Similarly, Scano’s study identified another type of polyols, mannitol, to be higher in FM [17].

## 4. Materials and Methods

### 4.1. Chemicals

All chemicals were purchased from Sigma-Aldrich (St. Louis, MO, USA). HPLC-grade methanol and acetonitrile were obtained from Merck Chemicals (Darmstadt, Germany). All aqueous solutions were prepared with ultrapure water produced by a Milli-Q system (18.2 MΩ, Millipore, Bedford, MA, USA).

### 4.2. Samples

HM samples were collected from 30 lactating mothers who exclusively breastfed their infants. The minimum sample size of 16 was required based on the calculation of MetaboAnalyst 3.0 (α set at 0.05 and a power of 80%) [63]. Non-inclusion criteria were preexisting maternal disease, fetal malformation and lactation failure. HM samples (10 mL foremilk) were collected by manual expression in the morning on the 42nd postpartum day. Sterile gloves were used; the first few drops (0.5–1 mL) were discarded; and the breast was thoroughly cleansed with chlorhexidine solution before manual collection. All of the samples were frozen at −80 °C before the analysis. Twenty whole BM samples, 10 PFM samples and 10 LFM samples were purchased from a local supermarket. The BM samples were selected from Brightdairy and Mengniu, who are the top two market sharers in China. The nutrient composition of Brightdairy BM is as follows: 1.24 g protein, 1.33 g fat, 1.95 g carbohydrate and 41.04 g calcium per 100 KJ. The nutrient composition of Mengniu BM is as follows: 1.12 g protein, 1.40 g fat, 1.68 g carbohydrate and 35.21 g calcium per 100 KJ. The PFM samples were from Wyeth S26 Gold 1st stage formula and Beingmate Championbaby 1st stage formula. The LFM samples were from Meadjohnson Enfamil ready-to-use 1st stage formula. Both the PFM and LFM samples were based on BM. The nutrient composition of PFM and LFM is listed in Table 2, and the ingredients adhere to the FM standard issued by the China Health Department [61]. This study was approved by the Institutional Review Board of Shanghai Jiao Tong University Affiliated Xinhua Hospital (XHEC-C-2012-017, 22 October 2012), in accordance with the principles of the Helsinki Declaration. Written informed consent was obtained from each participant.

### 4.3. Sample Preparation and Analysis by GC-TOFMS

Samples were derivatized and subsequently analyzed by GC-TOFMS, following our previously published protocols [64,65]. Briefly, 150 mg of PFM were dissolved in 1 mL of water according to the manufacturer’s instruction. An aliquot of 100 μL of the PFM solution, BM, HM and LFM was spiked with two internal standards (10 μL l-2-chlorophenylalanine in water, 0.3 mg/mL; 10 μL heptadecanoic acids in methanol, 1 mg/mL) and extracted with 300 μL of methanol and chloroform (3:1). After vortexing for 30 s, the extraction was kept at −20 °C for 10 min, then centrifuged at 12,000 rpm for 20 min, and an aliquot of 300 μL of supernatant was transferred to a glass sampling vial to vacuum dry at room temperature. The residue was derivatized using a two-step procedure. First, 80 μL of methoxyamine (15 mg/mL in pyridine) were added to the vial and kept at 30 °C for 90 min, followed by 80 μL of *N*,*O*-Bis(trimethylsilyl)trifluoroacetamide (BSTFA) (1% trimethylchlorosilane (TMCS)) at 70 °C for 60 min.

Each aliquot of 1 μL of the derivatized solution was injected, in splitless mode into an Agilent 6890N Gas Chromatograph, coupled with a Pegasus HT GC-TOFMS (LECO Corporation, St. Joseph, MI, USA). The samples were run in the order of BM-HM-LFM-PFM, alternately, to minimize systematic analytical deviations. Separation was achieved on a J&W DB-5MS capillary column (30 m × 250 µm I.D., 0.25-µm film thickness; Agilent Technologies, St. Clara, CA, USA). The temperature of injection, transfer interface and ion source was set to 270, 270 and 220 °C, respectively. The initial GC oven temperature was 80 °C; 2 min after injection, the GC oven temperature ramp was raised to 180 °C at a rate of 10 °C/min, to 230 °C at a rate of 6 °C/min, to 295 °C at a rate of 40 °C/min and maintained at 295 °C for 8 min. Helium was the carrier gas with a flow rate at 1 mL/min. A full scan mode (*m*/*z* 30–600) with the electron impact ionization at 70 eV was used. The mass fragments were acquired at a rate of 20 spectra/s in the TOFMS setting.

### 4.4. Sample Preparation and Analysis by UPLC-QTOFMS

Sample preparation and analysis using UPLC-QTOFMS was performed according to our previously published report [66]. Briefly, 150 mg of PFM were dissolved in 1 mL water according to the manufacturer’s instruction. An aliquot of 10 µL of the PFM solution, BM, HM and LFM was spiked with 20 μL of internal standard (l-2-chlorophenylalanine in water, 30 μg/mL) and extracted with 500 μL of acetonitrile and methanol (9:1). After vortexing for 2 min, the mixture was kept at −20 °C for 10 min and then centrifuged at 12,000 rpm for 20 min. The supernatant was transferred into the sampling vial pending UPLC-QTOFMS analysis. An aliquot of 5 µL of filtrate was injected in the order of BM-HM-LFM-PFM into a 100 mm × 2.1 mm, 1.7-μm BEH C_18_ column and held at 40 °C using an UPLC system (Waters Corp., Milford, MA, USA). Mobile Phase A consisted of water with 0.1% formic acid for positive (ES^+^) mode and water for negative (ES^−^) mode, whereas Mobile Phase B consisted of acetonitrile with 0.1% formic acid for ES^+^ and acetonitrile for ES^−^. The flow rate was 400 μL/min. The elution was a linear gradient of 1%–20% B over 0–1 min, 20%–70% B over 1–3 min, 70%–85% B over 3–8 min, 85%–100% B over 8–9 min and held at 100% B for 1 min. All of the samples were kept at 4 °C before injection.

The mass spectral data were collected using the Waters Xevo G1 QTOF (Waters Corp., Milford, MA, USA) equipped with an electrospray source operating in either ES^+^ or ES^−^. The source temperature was set at 120 °C with a cone gas flow of 50 L/h and a desolvation gas temperature of 350 °C with a desolvation gas flow of 650 L/h. In the case of the positive and negative ion mode, the capillary voltage was set to 3.2 kV and 3 kV with a cone voltage of 35 V and 50 V, respectively. Centroid data were collected from 50 to 1000 *m*/*z* with a scan time of 0.3 s and an inter-scan delay of 0.02 s over a 9.5-min analysis time. Leucine enkephalin was used as the lock mass (*m*/*z* 556.2771 in ES^+^ and 554.2615 in ES^−^) at a concentration of 100 ng/mL and a flow rate of 20 µL/min for all analyses.

### 4.5. Data Analyses

The acquired MS data from GC-TOFMS and UPLC-QTOFMS were analyzed according to our previously-published work [66]. The GC-TOF-MS data were analyzed by ChromaTOF software (version 4.34, LECO, Saint Joseph, MI, USA). After alignment with the Statistic Compare component, the *m*/*z* pairs, retention time (RT) and ion intensity of each compound were collected by the software and stored in a CSV file. The internal standard was used for data normalization. Any known pseudo-positive peak, such as peak caused by noise, column bleeding and BSTFA derivatization, was removed and excluded for future analysis.

The UPLC-QTOFMS dataset was analyzed by the MarkerLynx software (Waters Corp., Milford, MA, USA) using the following parameters. The mass range was set to be 50–1000 Da, the RT range to be 0–11 min and the mass tolerance to be 0.02 Da. The minimum intensity was set to be 15% of base peak intensity; the maximum mass per RT was set to be 6; the RT tolerance was set to be 0.1 min; and finally, the noise elimination level was set to be 10.00. The internal standard was used for data quality control and data normalization, and its peak was also deselected for analysis. The resulting dataset contained the information of the sample name, *m*/*z* pairs, RT and ion intensity. To obtain consistent differential variables, any peak with a missing value (ion intensity = 0) in more than 80% of samples was further removed from the dataset.

Metabolites’ identification was performed separately. GC-TOFMS metabolites were identified by matching the mass fragments with the standard mass spectral library (NIST 05) using the NIST MS Search Program (Version 2.0, NIST, Caithersburg, MD, USA), with a similarity threshold of ≥70%, and then verified manually by available reference standards in our lab. The metabolites obtained from the ES^+^ and ES^−^ modes of the UPLC-QTOFMS platform were identified by comparing the accurate mass, RT and characteristic *m*/*z* pairs with the available reference data from literatures. The Human Metabolome Database (http://www.hmdb.ca/) was used as an additional reference source for mass information.

### 4.6. Statistical Analysis

All of the annotated metabolites from the GC-TOFMS and UPLC-QTOFMS datasets were combined and imported into SIMCA-P 12.0 (Umetrics, Umeå, Sweden) for multivariate statistical analysis. Partial least squares-discriminant analysis (PLS-DA) was performed to visualize the variations among the different milk types. Models were tested by 7-fold cross-validation using *R*^2^ and *Q*^2^ parameters, where *R*^2^ represents the goodness of fit and *Q*^2^ represents the goodness of prediction. A permutation test (100 tests) was performed to test the validity of PLS-DA model. The variable importance in the projection (VIP) values of all of the metabolites obtained from the PLS-DA model were taken as a criterion for differential metabolites selection. Those variables with a VIP of more than 1.0 were considered relevant for group discrimination [67]. The Mann–Whitney U test was used to measure the significance of each metabolite. Metabolites responsible for the differentiation of them could be identified by comprehensive consideration of these two coefficients (VIP > 1, *p* < 0.05). Heatmaps of the identified metabolites were generated using the Heatmap Illustrator 1.0 (The CUCKOO Workgroup, Wuhan, China) and were further analyzed using agglomerative hierarchical cluster analysis based on Pearson correlation distances.

To compare the abundance of selected differential metabolites between three milk types, the intensity of each metabolite was represented as the median (interquartile range). The values below the limit of detection (LOD) were substituted with LOD/2 before statistical analysis [68]. All variables were assessed by the Kruskal-Wallis non-parametric H test followed by the Bonferroni-corrected post-hoc test. A *p*-value <0.05 was considered to be statistically significant. All statistical analyses were computed using IBM SPSS Statistics for Windows, Version 19.0 (IBM, Armonk, New York, NY, USA).

## 5. Conclusions

The global comparison of small molecule metabolites among HM, FM and BM may help improve the nutritional quality of FM. However, despite improving its quality, FM cannot totally mimic the biological impact of HM. Our study represents a thorough metabolomic analysis of these three milk types. The results indicate that HM, FM and BM have their own unique metabolic profile. Several distinct metabolite groups, including non-esterified fatty acids, free amino acids, TCA intermediates and free carbohydrates, separate HM from FM and BM. Higher levels of non-esterified SFAs with aliphatic tails <16 carbons, MUFAs and PUFAs, and lower levels of TCA intermediates are characteristic of HM, as compared with FM and BM. The content of non-esterified fatty acids may reflect the hydrolysis of triglycerides in different milk types.

## Figures and Tables

**Table 1:** Reported subject characteristics.

Perinatal Characteristics ^1^	
**Maternal**	
Age (years)	29.47 ± 4.37 (22, 47)
Pre-pregnant BMI (kg/m^2^)	20.73 ± 3.23 (17.42, 34.17)
Multiparity (%)	13.3
C-section (%)	46.7
Gestational weeks (W)	39.71 ± 0.64 (38.6, 41.2)
**Infant**	
Birth weight (g)	3340 ± 388 (2700, 4325)
Male (%)	54.0

^1^ Continuous variables are represented as the mean ± SD (range). Discontinuous variables are represented as frequencies per total sample size. *n* = 30.

**Table 2:** The nutrient composition of the powdered formula milk (PFM) and liquid formula milk (LFM) evaluated in this study.

Nutrients per 100 KJ	Unit	PFM (Wyeth)	PFM (Beingmate)	LFM (Meadjohnson)
Protein	g	0.48	0.57	0.49
Carbohydrate	g	2.55	2.60	2.68
Fat	g	1.29	1.25	1.29
prebiotics	g	0.11	0.03	Not quantified
Linoleic acid	mg	185.63	210.00	239.12
Linolenic acid	mg	15.00	21.00	Not quantified
DHA	mg	4.50	1.30	Not quantified
AA	mg	4.50	2.60	Not quantified
Vitamin A	IU	86.26	72.33	71.74
Vitamin D	IU	17.12	12.74	17.93
Vitamin E	IU	0.31	0.22	0.36
Vitamin K	µg	2.39	2.40	1.91
Vitamin C	mg	3.21	3.30	2.15
Vitamin B1	µg	44.63	22.90	23.91
Vitamin B2	µg	39.68	29.60	35.87
Vitamin B6	µg	26.77	13.30	14.35
Vitamin B12	µg	0.08	0.05	0.06
Folic acid	µg	3.21	3.30	3.59
Biotin	µg	0.89	0.57	1.05
Pantothenic acid	µg	133.91	148.97	107.60
Niacin	µg	219.99	182.93	251.08
Inositol	mg	1.61	1.30	1.12
Choline	mg	5.85	3.70	3.83
Calcium	mg	15.28	18.10	18.65
Phosphorus	mg	8.57	11.20	10.04
Iron	mg	0.29	0.20	0.43
Copper	µg	18.14	11.20	21.52
Zinc	mg	0.21	0.19	0.18
Iodine	µg	3.57	3.40	1.43
Selenium	µg	0.72	0.70	0.48

## Supplementary Materials

Supplementary materials can be found at www.mdpi.com/1422-0067/17/12/2128/s1.

## Abbreviations

GC-TOFMS	gas chromatography time-of-flight mass spectrometry
UPLC-QTOFMS	ultra-performance liquid chromatography quadrupole time-of-flight mass spectrometry
HM	human breast-milk
HMOs	human milk oligosaccharides
BMOs	bovine milk oligosaccharides
FM	formula milk
BM	bovine milk
TCA	tricarboxylic acid
PFM	powdered formula milk
LFM	liquid formula milk
PLS-DA	partial least squares-discriminant analysis
NEFAs	non-esterified fatty acids
SFAs	saturated fatty acids
MUFAs	monounsaturated fatty acids
PUFAs	polyunsaturated fatty acids; FAAs: free amino acids
