# Supplementary material for: Metabolomic Approaches to Explore Chemical Diversity of Human Breast-Milk, Formula Milk and Bovine Milk
Source: Int J Mol Sci. 2016 Dec 17;17(12):2128. doi: 10.3390/ijms17122128 (PMC5187928; doi:10.3390/ijms17122128)
Supplement: Supplementary file 1 [file ijms-17-02128-s001.pdf]

Supplementary Materials: Metabolomic Approaches to Explore Chemical Diversity of Human Breast-Milk, Formula Milk and Bovine Milk

Linxi Qian, Aihua Zhao, Yinan Zhang, Tianlu Chen, Steven H. Zeisel, Wei Jia and Wei Cai

Table S1. The differential metabolites obtained from multivariate and univariate analysis of formula milk relative to human breast milk.

Metabolites	Platform [a]	VIP [b]	FC [c]	*p*-Value [d]	Class
5-oxoproline	G	1.50	0.06	2.83×10^{-9}	amino acid
alanine	G	1.62	0.06	2.83×10^{-9}	amino acid
phenylalanine	G	1.7	32.91	1.96×10^{-11}	amino acid
glutamine	G	1.56	0.001	8.24×10^{-10}	amino acid
arginine	P	1.23	0.12	1.99×10^{-6}	amino acid
glutamic acid	G	1.61	0.15	3.98×10^{-7}	amino acid
glycine	G	1.32	0.17	2.83×10^{-9}	amino acid
histidine	P	1.18	2.40	1.74×10^{-5}	amino acid
serine	G	1.54	0.03	2.51×10^{-9}	amino acid
tyrosine	P	1.61	0.001	4.78×10^{-8}	amino acid
taurine	N	1.45	1.91	1.04×10^{-4}	amino acid
valine	G	1.55	0.05	2.63×10^{-8}	amino acid
acetylglucosamine	P	1.42	2.15	6.97×10^{-7}	carbohydrate
acetylneuraminic acid	N	1.27	0.44	3.97×10^{-7}	carbohydrate
arabinose	N	1.13	7.43	6.27×10^{-8}	carbohydrate
arabitol	G	1.32	0.35	5.59×10^{-7}	carbohydrate
fructose	G	1.42	30.63	3.20×10^{-5}	carbohydrate
fucose	G	1.21	0.04	9.99×10^{-8}	carbohydrate
glucosamine	N	1.16	1.81	2.82×10^{-9}	carbohydrate
glycerol	G	1.17	0.16	2.94×10^{-8}	carbohydrate
glyceraldehyde	N	1.26	1.51	3.26×10^{-6}	carbohydrate
maltose	G	1.31	5.06	4.42×10^{-7}	carbohydrate
threitol	P	1.29	7.22	9.47×10^{-7}	carbohydrate
xylose	P	1.06	0.16	5.35×10^{-4}	carbohydrate
arachidonic acid	N	1.47	ND [e]	7.70×10^{-16}	fatty acid
capric acid	G	1.51	ND	1.65×10^{-14}	fatty acid
dihydroxypropanoic acid	G	1.01	0.59	2.04×10^{-5}	fatty acid
docosadienoic acid	N	1.62	ND	3.91×10^{-19}	fatty acid
docosahexaenoic acid	P	1.42	ND	1.15×10^{-9}	fatty acid
docosapentaenoic acid	N	1.38	ND	6.59×10^{-11}	fatty acid
eicosadienoic acid	G	1.37	ND	1.07×10^{-10}	fatty acid
eicosapentaenoic acid	N	1.06	ND	5.72×10^{-6}	fatty acid
eicosatrienoic acid	G	1.65	ND	5.02×10^{-21}	fatty acid
eicosenoic acid	G	1.14	ND	7.38×10^{-7}	fatty acid
lauric acid	G	1.53	ND	5.27×10^{-15}	fatty acid
linoleic acid	N	1.50	0.05	6.91×10^{-12}	fatty acid
α-linolenic acid	N	1.50	ND	2.80×10^{-14}	fatty acid
γ-linolenic acid	N	1.47	ND	8.32×10^{-13}	fatty acid
myristic acid	N	1.23	ND	3.68×10^{-8}	fatty acid
myristoleic acid	G	1.18	ND	2.31×10^{-7}	fatty acid
oleic acid	G	1.25	ND	2.57×10^{-8}	fatty acid

Table S1. *Cont.*

Metabolites	Platform [a]	VIP [b]	FC [c]	*p*-Value [d]	Class
palmitic acid	G	1.16	0.02	2.47×10^{-5}	fatty acid
palmitoleic acid	P	1.59	ND	1.04×10^{-17}	fatty acid
pentadecanoic acid	N	1.53	ND	2.83×10^{-16}	fatty acid
tridecanoic acid	G	1.15	ND	4.30×10^{-7}	fatty acid
2-ketoglutaramic acid	G	1.28	26.24	3.03×10^{-5}	TCA cycle
2-ketoglutaric acid	G	1.21	4.23	9.17×10^{-6}	TCA cycle
citric acid	G	1.43	11.56	1.70×10^{-10}	TCA cycle
fumaric acid	G	1.66	28.54	1.05×10^{-8}	TCA cycle
adenine	P	1.13	21.15	1.54×10^{-9}	vitamin
carnitine	G	1.39	4.19	2.61×10^{-8}	vitamin
lipoic acid	P	1.47	0.10	4.90×10^{-8}	vitamin
niacinamide	P	1.45	32.38	4.59×10^{-4}	vitamin
orotic acid	G	1.28	12.58	7.09×10^{-8}	vitamin
pantothenic acid	N	1.15	1.81	1.73×10^{-6}	vitamin
glycocholic acid	P	1.41	0.11	2.29×10^{-7}	bile acid
hydroxycholic acid	P	1.57	0.10	3.65×10^{-8}	bile acid
4-methoxycinnamic acid	P	1.36	4.37	4.05×10^{-9}	other
acetylcystathionine	P	1.49	0.09	1.12×10^{-7}	other
aldosterone	P	1.35	0.16	2.28×10^{-6}	other
urea	G	1.84	22.18	2.79×10^{-9}	other

[a] G: data from GC-TOFMS; N: data from UPLC-QTOFMS in negative mode; P: data from UPLC-QTOFMS in positive mode; [b] VIP (variable importance in projection) score from PLS-DA model; [c] FC (fold change) of different metabolites in formula milk compared to human breast milk; [d] *p*-value from the Mann–Whitney U test; [e] below the limit of detection.

Table S2. The differential metabolites obtained from multivariate and univariate analyses of bovine milk relative to human breast milk.

Metabolites	Platform [a]	VIP [b]	FC [c]	*p*-Value [d]	Class
5-oxoproline	G	1.39	0.32	3.19×10^{-9}	amino acid
alanine	G	1.37	0.36	7.18×10^{-8}	amino acid
arginine	P	1.24	0.04	6.92×10^{-8}	amino acid
glutamic acid	G	1.52	0.35	2.83×10^{-9}	amino acid
glutamine	G	1.29	0.18	1.90×10^{-7}	amino acid
phenylalanine	G	1.42	25.79	1.82×10^{-11}	amino acid
proline	G	1.22	2.3	7.39×10^{-7}	amino acid
serine	G	1.55	0.13	2.82×10^{-9}	amino acid
taurine	N	1.33	0.10	2.82×10^{-9}	amino acid
tyrosine	P	1.36	0.001	4.78×10^{-8}	amino acid
valine	G	1.31	0.34	4.81×10^{-8}	amino acid
3′-ketolactose	N	1.26	3.30	6.68×10^{-7}	carbohydrate
4-ketoglucose	G	1.35	0.18	9.52×10^{-8}	carbohydrate
acetylglucosamine	P	1.74	12.64	4.92×10^{-9}	carbohydrate
allofuranose	G	1.35	17.07	2.49×10^{-9}	carbohydrate
arabinose	N	1.13	3.36	3.30×10^{-6}	carbohydrate
fucose	G	1.41	0.11	5.77×10^{-6}	carbohydrate
glucosamine	N	1.22	1.56	4.95×10^{-9}	carbohydrate
glyceraldehyde	N	1.04	1.97	4.97×10^{-9}	carbohydrate
maltose	G	1.13	0.20	2.04×10^{-4}	carbohydrate

Table S2. *Cont.*

Metabolites	Platform [a]	VIP [b]	FC [c]	*p*-Value [d]	Class
mannose	G	1.35	16.38	4.97×10^{-9}	carbohydrate
ribose	G	1.34	1.44	2.60×10^{-7}	carbohydrate
arachidonic acid	N	1.58	ND [e]	8.70×10^{-19}	fatty acid
capric acid	G	1.49	0.04	5.51×10^{-15}	fatty acid
docosadienoic acid	N	1.62	ND	3.91×10^{-19}	fatty acid
docosahexaenoic acid	P	1.24	ND	1.01×10^{-8}	fatty acid
docosapentaenoic acid	N	1.35	ND	7.68×10^{-11}	fatty acid
eicosadienoic acid	G	1.35	ND	8.23×10^{-9}	fatty acid
eicosapentaenoic acid	N	1.04	ND	5.72×10^{-6}	fatty acid
eicosatrienoic acid	G	1.61	ND	8.93×10^{-21}	fatty acid
eicosenoic acid	G	1.09	0.04	1.47×10^{-6}	fatty acid
lauric acid	G	1.32	0.04	4.21×10^{-9}	fatty acid
linoleic acid	N	1.72	ND	7.12×10^{-15}	fatty acid
α-linolenic acid	N	1.47	ND	3.18×10^{-14}	fatty acid
γ-linolenic acid	N	1.69	ND	8.32×10^{-13}	fatty acid
myristic acid	N	1.44	0.17	2.83×10^{-13}	fatty acid
myristoleic acid	G	1.10	0.07	1.21×10^{-6}	fatty acid
oleic acid	G	1.27	ND	1.89×10^{-8}	fatty acid
palmitic acid	G	1.33	31.87	3.41×10^{-15}	fatty acid
palmitoleic acid	P	1.55	ND	2.54×10^{-17}	fatty acid
pentadecanoic acid	N	1.23	0.44	1.47×10^{-8}	fatty acid
stearic acid	G	1.18	3.15	4.77×10^{-6}	fatty acid
tridecanoic acid	G	1.13	ND	4.30×10^{-7}	fatty acid
2-ketoglutaramic acid	G	1.44	28.24	5.21×10^{-9}	TCA cycle
cis-aconitic acid	G	1.62	4.08	4.97×10^{-9}	TCA cycle
citric acid	N	1.65	15.07	4.96×10^{-9}	TCA cycle
fumaric acid	G	1.62	34.53	2.29×10^{-10}	TCA cycle
malic acid	G	1.70	13.05	4.97×10^{-9}	TCA cycle
biotin	P	1.49	2.79	4.97×10^{-9}	vitamin
lipoic acid	P	1.40	0.09	4.95×10^{-9}	vitamin
orotic acid	G	1.26	10.78	1.14×10^{-2}	vitamin
tocopherol	N	1.23	0.01	3.53×10^{-7}	vitamin
glycocholic acid	P	1.44	0.03	1.32×10^{-8}	bile acid
hydroxycholic acid	P	1.47	0.03	2.33×10^{-8}	bile acid
2-ethoxy-acetic acid	N	1.52	0.005	1.98×10^{-9}	other
2-indolecarboxylic acid	N	1.64	0.78	4.97×10^{-9}	other
3-aminopropionaldehyde	N	1.45	1.67	7.15×10^{-9}	other
3-hydroxybutanoic acid	G	1.58	8.71	3.39×10^{-8}	other
4-methoxycinnamic acid	N	1.74	16.96	4.96×10^{-9}	other
cholesterol	G	1.40	2.36	8.53×10^{-8}	other
creatinine	P	1.57	2.23	4.97×10^{-9}	other
indole-5,6-quinone	N	1.42	0.57	7.19×10^{-9}	other
methylenebutanedioic acid	G	1.10	1.44	1.03×10^{-5}	other
pipecolic acid	G	1.53	3.32	8.05×10^{-9}	other
uracil	G	1.52	2.20	4.97×10^{-9}	other
uridine	G	1.23	2.61	9.81×10^{-7}	other

[a] G: data from GC-TOFMS; N: data from UPLC-QTOFMS in negative mode; P: data from UPLC-QTOFMS in positive mode; [b] VIP (variable importance in projection) score from PLS-DA model; [c] FC (fold change) of different metabolites in bovine milk compared to human breast milk; [d] *p*-value from the Mann–Whitney U test; [e] below the limit of detection.
